# Protocol for preliminary, multicenteric validation of “PoCOsteo device”: A point of care tool for proteomic and genomic study of osteoporosis

**DOI:** 10.1093/biomethods/bpae006

**Published:** 2024-03-22

**Authors:** Farideh Razi, Afshin Ostovar, Noushin Fahimfar, Mahsa M. Amoli, Saeed Ebrahimi Fana, Hans Peter Dimai, Barbara Obermayer-Pietsch, Barbara Luegger, Fernando Rivadeneira, Iraj Nabipour, Bagher Larijani, Patricia Khashayar

**Affiliations:** Metabolomics and Genomics Research Center, Endocrinology and Metabolism Molecular-Cellular Sciences Institute, Tehran University of Medical Sciences, Tehran, Iran; Osteoporosis Research Center, Endocrinology and Metabolism Clinical Sciences Institute, Tehran University of Medical Sciences, Tehran, Iran; Osteoporosis Research Center, Endocrinology and Metabolism Clinical Sciences Institute, Tehran University of Medical Sciences, Tehran, Iran; Metabolic Disorders Research Center (MDRC), Endocrinology and Metabolism Molecular-Cellular Sciences Institute, Tehran University of Medical Sciences (TUMS), Tehran, Iran; Department of Clinical Biochemistry, Tehran University of Medical Sciences, Tehran, Iran; Department of Internal Medicine, Division of Endocrinology and Diabetology, Medical University of Graz, Graz, Styria, Austria; Department of Internal Medicine, Division of Endocrinology and Diabetology, Medical University of Graz, Graz, Styria, Austria; Department of Internal Medicine, Division of Endocrinology and Diabetology, Medical University of Graz, Graz, Styria, Austria; Erasmus Universitair Medisch Centrum Rotterdam, Rotterdam, The Netherlands; The Persian Gulf Marine Biotechnology Research Center, The Persian Gulf Biomedical Sciences Research Institute, Bushehr University of Medical Sciences, Bushehr, Iran; Endocrinology and Metabolism Research Center, Endocrinology and Metabolism Clinical Sciences Institute, Tehran University of Medical Sciences, Tehran, Iran; Center for Microsystems Technology, Imec & Ghent University, Zwijnaarde, Gent, Belgium

**Keywords:** osteoporosis, validation studies, proteomic, genomics

## Abstract

One of the goals of the HORIZON 2020 project PoCOsteo was to develop a medical device, which would measure and/or quantify proteomic as well as genomic factors as present in whole blood samples collected through finger prick. After validating the tool in the clinical setting, the next step would be its clinical validation based on the existing guidelines. This article presents the protocol of a validation study to be carried out independently at two different centers (Division of Endocrinology and Diabetology at the Medical University of Graz as a clinic-based cohort, and the Endocrinology and Metabolism Research Institute at the Tehran University of Medical Sciences as a population-based cohort). It aims to assess the tool according to the Clinical & Laboratory Standards Institute guidelines, confirming if the proteomics and genomics measurements provided by the tool are accurate and reproducible compared with the existing state-of-the-art tests. This is the first time that such a detailed protocol for lab validation of a medical tool for proteomics and genomic measurement is designed based on the existing guidelines and thus could be used as a template for clinical validation of future point-of-care tools. Moreover, the multicentric cohort design will allow the study of a large number of diverse individuals, which will improve the validity and generalizability of the results for different settings.

## Background

Osteoporosis is a widespread disease associated with low bone mineral density (BMD), microarchitectural decay of bone tissue, and bone fragility [1]. Bone turnover markers (BTMs) can be used to monitor patients’ responses to treatment for osteoporosis and are also considered potential predictors of fracture risk [2, 3]. The International Osteoporosis Foundation (IOF) and the International Federation of Clinical Chemistry and Laboratory Medicine (IFCC) suggest that the combination of a bone formation marker (serum procollagen type I N-terminal propeptide, PINP) and a bone resorption marker (serum C-terminal telopeptide of type I collagen, CTx) to be used as reference analytes for BTM measurements in clinical studies [4]. Furthermore, several countries have included such measurements in their national guidelines, mostly for osteoporosis treatment monitoring [5–9].

The rationale behind the changes in BTM serum levels in the course of osteoporosis disease and the treatment phase has been widely investigated among postmenopausal women. Menopause-related estrogen deficiency and other causes of osteoporosis results in a general increase in bone remodeling and, therefore, an imbalance in bone formation and resorption [10–12]. Osteoporosis therapy, on the other hand, triggers rapid and significant changes in BTMs with many studies confirming changes in PINP and CTx serum levels within a short time after the start of the treatment [13–19]. In this regard, treatment with anti-resorptive agents is associated with an early decrease, within the first 3 months, in bone resorption markers followed by a decrease in bone formation markers. In contrast, an anabolic agent, such as teriparatide, initially causes an increase in bone formation markers and a subsequent surge in bone resorption markers [20]. A recently approved medication, romosozumab (a humanized monoclonal antibody IgG2 against sclerostin), increases bone formation and suppresses bone resorption, leading to a transient increase in bone formation markers and a more sustained decrease in bone resorption ones [21, 22]. Of note, changes in BMD, as measured by DXA, are less prominent during therapy and correlate less closely to the fracture risk reduction than that of BTMs, especially with anti-resorptive treatment [23].

There are several assays for CTx and P1NP measurement on the market; the most frequently used of which are enzyme-linked immunosorbent assay (ELISA), automated chemiluminescence (CLIA) from Immunodiagnostic Systems, electrochemiluminescent immunoassay (ECLIA) from Roche Diagnostics, and radioimmunoassay-based assays by Orion Diagnostica [24]. Other approaches like multiplex miniaturized chip format assays and biosensor assays have also been investigated, but not commercialized until this point [25, 26].

On the other hand, genetic studies of candidate genes as well as genome-wide association studies (GWAS) have documented associations between single-nucleotide polymorphisms (SNPs) in bone-relevant genes and BMD [27, 28]. By 2019, more than 20 primary GWAS and GWAS meta-analyses have identified hundreds of associations in this regard. BMD and fracture risk were the principal targets of GWASs, mainly due to its strong correlation with fracture, high heritability, and relative ease of evaluation in large cohorts, but fracture risk is another important component in genetic risk scores. SNP detection technologies are currently used to search for new polymorphisms and identify alleles in target sequences of known polymorphisms [29]. As the cost of SNP detection continues to decline, even very ambitious studies might become economically accessible [30]. Therefore, choosing a relevant SNP panel with an appropriate polygenic risk score (PRS) could improve the definition of fracture risk with reasonable cost [31–40].

To answer these needs, the PoCOsteo device was built. This is an in-office tool that brings together biomarker measurement and profiling of genetic variations together, enabling physicians to have the test results during the individual consultation time of a patient, enhance the predictive accuracy of fracture prognosis (commonly calculated through FRAX) and provide patients with personalized care [41, 42]. The device is designed to work with two cartridges, a proteomic cartridge designed to measure blood levels of two BTM (CTX and P1NP) and a genomic cartridge designed to study the presence of certain fracture-related SNPs [WNT16 (rs2908007) RSPO3 (rs10457487), RNMT/FAM210A (rs4635400), SOST (rs2741856), MCM6/LCT-13910 (rs4988235)] in capillary blood [40]. The cartridges are designed to work using fingerprick blood and provide the results in a very short time.

## Objectives

The PoCOsteo study is an EU-funded Horizon 2020 project designed to provide adequate parameters for fracture risk identification and treatment monitoring while increasing a patient’s access to laboratory values. The point-of-care (PoC) device is created and built up for P1NP and CTx measurement and profiling of genetic variations (mentioned above) using capillary blood. The device supports the estimation and evaluation of fracture risk according to the ASSURED criteria (affordable, sensitive, specific, user-friendly, rapid and robust, equipment-free, and deliverable to users) [43]. This method enhances personalized care among osteoporosis patients via the application of innovative equipment even in remote areas.

This protocol briefly explains how manufacturers preliminary evaluated and validated the device in a multicenter study to provide initial data required to fulfill the requirements set out in the IVD Regulation 2017/746 [44].

## Methods/design

### Study design

The device will be validated using various experiments to be described later on in this article by applying standard control material and biological samples from both healthy and osteoporosis patients. In certain cases, it is possible to combine tests by performing several experiments simultaneously or using the results of a single test for multiple purposes, with the aim of saving cost and time. The “Analytical Performance and Clinical Studies” section in the following provides an outline of the tests to be done to validate the proteomic and genomic aspects of the tool, independently.

### Study population

The study participants will be selected from among patients recruited in the PoCOsteo clinical study at MUG and EMRI [42]. In case additional patients are required, the inclusion criteria will be the same as the PoCOsteo clinical study. A venous whole blood sample is obtained from each participant for analysis and storing in the biobank. As the sample of choice for the PoCOsteo device is whole capillary blood, capillary blood will be collected from a subgroup of the participants through fingerpricks. Sample selection is random unless particular criteria are needed.

### Ethics and dissemination

The study will be conducted according to the World Medical Association Declaration of Helsinki and approved by the Research Ethics Committee of EMRI, Tehran University of Medical Sciences (IR. TUMS. EMRI.REC.1395.00152) and Ethical Committee of the Medical University Graz.

### Analytical performance and clinical studies

#### Proteomics section

The first step is to assess the accuracy of the device by examining two cartridges with different analytes for 5 consecutive days. Then the device’s capability to accurately measure CTX and P1NP will be evaluated [45]. The number of tests needed for each of the following items will be determined by the CLSI guidelines [46].

- Precision of measurement: Precision is defined as “the closeness of agreement between independent test results obtained under stipulated conditions” [47]. To assess precision, therefore, at least two stable samples, which simulate the characteristics of real clinical samples with different serum levels of CTx and P1NP markers, ideally near their medical decision levels, will be tested in duplicate in a minimum of 20 working days, two runs per day. This leads to 80 test results for each sample (20 days × 2 runs × 2 replicates). An alternative approach is to analyze the same samples five times in three centers for 5 consecutive days (5 days × 5 replicates/center). The data from the familiarization period can be incorporated with the final data only if the results are consistent. The data will be checked for outliers. Thereafter, the repeatability and reproducibility (expressed as coefficient variation %) will be calculated according to the CLSI EP05-A3 guidelines [46].
- Trueness of measurement: Trueness on the other hand is defined as “the closeness of agreement between the average value obtained from a large series of test results and an accepted reference value” [47]. The new and state-of-the-art methods will be compared to determine the trueness of the new tool. Ideally, a reference method with low bias and imprecision values is used for such comparison studies. In the absence of a reference method, such as in this case, commercial methods can be used as comparative methods. As a result, the Roche electrochemiluminescence method will be adopted as the reference method. In this regard, 40 clinical samples with different concentrations of the analytes (within the measuring range of the method) will be analyzed simultaneously in duplicates using both the PoCOsteo device and the Roche system. The comparison of the results will be illustrated as scatter and difference plots, and then the bias will be calculated based on the CLSI EP09 A3 techniques [48].
- Measurement range: Samples from a group of patients with the analyte concentration near the expected upper reportable limit (or samples spiked by the analyte to reach the upper limit) will be diluted with a suitable diluent (e.g. Roche diluent). To establish the linear range, 11 samples with target levels 20%–30% higher than the expected measuring range will be prepared. Each diluted sample will be tested in duplicate. Results will be plotted and then the range of the acceptable linear response will be calculated statistically according to the CLSI EP06-A guidelines [49].
- Analytical sensitivity: This term refers to the assay’s ability to detect very low concentrations of a given substance in a biological specimen [50]. The limit of blank (LoB) will be estimated based on the repeated analysis of blank samples; then mean and mean + 2SD will be considered as LoB and limit of detection (LoD), respectively. The limit of quantification will be estimated based on the repeated examination of predefined low-concentration samples. Total error (TE) will be calculated from the measurement results. If the TE for each reagent lot meets the predetermined goal, the mean concentration will be reported as the limit of quantification (LoQ) [51, 52].
- Analytical specificity: This term refers to “the ability of the bioanalytical method to measure and differentiate the analytes in the presence of other possible components” [53, 54]. According to the materials and reagents used in the device, relevant endogenous and exogenous materials likely to interfere with the measurements will be checked. In this regard, the effect of common preanalytical abnormalities (hemolysis, icterus, and lipemia), prescription and over-the-counter medications commonly prescribed in osteoporosis patients (Zoledronate, Ibandronate, Alendronate, Risedronate, calcium, and vitamin D), and analytes such as osteocalcin, PTH, bone ALP will be assessed. A pool of fresh specimens from healthy individuals not taking any medications (control sample) will be collected. As many of the aforementioned factors already exist in healthy individuals, these specimens will be spiked with higher-than-normal concentrations of these interfering substances (test samples). The control and test samples will then be examined using the device, and the results will be interpreted based on the total acceptable error for each analyte [55].
- Reference interval: The reference interval for CTx and PINP must be confirmed by examining a minimum of 20 reference subjects for each group (men over 50 years, pre- and postmenopausal women). The results will be analyzed based on the CLSI EP28-A3c [56]. Data collected during the trueness assessment and clinical studies can be used for this purpose.

####  

Further clinical studies will be designed to ensure the device’s ease of use by the healthcare personnel and to evaluate its capability in detecting the least significant change (LSC) in BTM levels using samples of individuals with newly diagnosed osteoporosis (40 individuals). The samples will be tested by both the PoCOsteo device and Roche before and after receiving treatment. The type of medications, the follow-up time, and the time of second blood sampling will be defined based on a local protocol in the respective study clinic. BMD will be measured at baseline (before the start of treatment). The patients could be selected from those already enrolled in PoCOsteo or additional patients using similar inclusion and exclusion criteria as the PoCOsteo clinical study [42].

#### Genomics section

After getting familiar with the instrument by examining control material or frozen/fresh whole blood samples with different genotypes, accuracy studies are performed. The genomic profile of individuals does not change over time; thus, the analytical performance and clinical studies could be integrated regardless of whether samples used in the analytical performance studies are collected from healthy people or osteoporosis patients.

The comparison studies compare the results of the PoCOsteo results with that of the reference method using frozen and fresh blood samples. Forty samples with known genotypes, homozygous (wild type and mutant) and heterozygous for each SNP including WNT16 (rs2908007) RSPO3(rs10457487), RNMT/FAM210A(rs4635400), SOST (rs2741856), MCM6/LCT-13910 (rs4988235), previously tested using TaqMan SNP genotyping assays from Applied Biosystems will be tested using the PoCOsteo device. For this purpose, forward and reverse primers to perform Sanger sequencing and PCR amplification of each region containing specific SNP will be designed for each SNP separately. If the results do not match for a sample, the experiments will be repeated, and duplicate tests will be checked for similarity if necessary. The results are also analyzed to calculate the sensitivity and specificity of the test.

#### Outcomes

The accomplishment of the above-mentioned experiments provides us with sufficient information about the following specifications:

Proteomics part: precision (CV%), trueness (Bias%), measurement range, LoD, LoQ, interfering substances, reference interval, LSC.

Genomics part: specificity, sensitivity.

## Discussion

PoCOsteo tool designed to reduce the analytical variability of BTMs and integrate the BTM levels with genetic profiling of each individual will be validated. This aims to overcome the challenges faced by clinicians in performing these tests: Although the IOF and IFCC suggest PINP and CTx as the reference analytes for the BTM measurements, their clinical application is limited due to preanalytical and analytical variability [4]. As a result, the harmonization of the assays is necessary to reduce the discrepancy noted between the results obtained from different assays [45].

Determination of the analytical specification goal is an important issue for harmonizing the assays. In the European Biological Variation Study, Cavalier *et al*. calculated the desirable imprecision (CV%) and bias based on within-subject (CVI) and between-subject (CVG) variability as follows:

CV analytic= 12 CV I Bias = 0.25CVI2+CVG2

According to their calculations, the desirable imprecision and bias for CTx were 7.6% and 12.6%, and for P1NP was 4.4% and 9.2%, respectively [11]. This is while some studies on available kits in the market, such as the Trio study, have failed to meet these goals [57]. The same results were obtained in a multicenter evaluating three popular commercial kits, including Roche Diagnostics, Immunodiagnostic systems, and Orion Siagnostic (aidian) for PINP measurement [52]. They calculated the median of biological variation generated from various studies and then the desirable and minimum CV% as 0.5 CVI and 0.75 CVI. These values were 3.7% and 5.5%, respectively. The CV% for Roche diagnostics ranged between 3.1% and 5.2%, for IDS 2.3% and 8.4%, and for Orion diagnostic 7.1% and 20.5%. In another study by the same authors, the CV% for CTX assays from Roche Diagnostics, Immunodiagnostic Systems (ELISA & CLIA) were calculated to be less than 8.2%, 12.2%, and 10.4%, respectively [58].

Since the ECLIA method from Roche Diagnostics is considered a reference method in this protocol, the results of the PocOsteo device will be assumed acceptable if they are not only within the acceptable imprecision and trueness range but also comparable with that of Roche Diagnostics.

There is no specific standard for the reportable range and interfering substances; the values reported by other commercial assays are, therefore, considered as the reference for the current protocol. The measuring range for PINP and CTx is expected to be approximately 0.7–3.4 µg/dl and 5–250 µg/l, respectively. Any change more than ±10% of the initial value in the presence of the interfering substances is considered as interference.

Despite the high number of studies on the development and evaluation of PoC devices for genetic identification of pathogens, only a few publications are dedicated to human genetic studies, none in the field of osteoporosis [38, 39]. As far as we know, the PoCOsteo device is the first PoC tool capable of performing genotyping for osteoporosis risk determination. Its results and characteristics, therefore, could not be directly compared with any of the available tools. However, considering the mentioned publications, it is necessary to reach a level of 100% concordance between the results of the Pocosteo device and the routinely used TaqMan methods.

In conclusion, the PoCOsteo device opens up new avenues for individualized, though quick and reliable testing of relevant BTMs and candidate genes in diagnosis and monitoring of osteoporosis patients.

## Strengths and limitations

This is the first time that such a detailed protocol for lab validation of a medical tool for proteomics and genomic measurement is designed based on the existing guidelines and thus could be used as a template for clinical validation of future PoC tools. Moreover, the multicentric cohort design will allow the study of a large number of diverse individuals, which will improve the validity and generalizability of the results for different settings.

However, the difference in the nature of the study populations could result in some disparities in the results. As the subjects are collected from a single center in each continent and the tests are performed in a single center from a teaching hospital, the generalizability of the results to the whole European and Middle Eastern population might not be possible. Considering the diversity of the confounding factors, taking all of them into account during the validation studies might not be feasible. Moreover, the fact that mass production of the tool and cartridges is not yet possible could limit the number of cartridges to be produced during the study period and thus the tests to be done in each section. However, as mentioned earlier, combining the tests can help guarantee the required cartridges for each test parameter.

## Ethics approval and consent to participate

The study is conducted according to the World Medical Association Declaration of Helsinki and approved by the Research Ethics Committee of EMRI, Tehran University of Medical Sciences (IR. TUMS. EMRI.REC.1395.00152) and Ethical Committee of the Medical University Graz.

## Consent for publication

All the authors have given their consent for the publication of the article

## Availability of data and material

Data can be available where applicable.

*Conflict of interest statement*. No conflict of interest.
